# Multilocus sequence typing of *Streptococcus thermophilus* from naturally fermented dairy foods in China and Mongolia

**DOI:** 10.1186/s12866-015-0551-0

**Published:** 2015-10-26

**Authors:** Jie Yu, Zhihong Sun, Wenjun Liu, Xiaoxia Xi, Yuqin Song, Haiyan Xu, Qiang Lv, Qiuhua Bao, Bilige Menghe, Tiansong Sun

**Affiliations:** Key Laboratory of Dairy Biotechnology and Engineering, Education Ministry of P. R. China, Inner Mongolia Agricultural University, Hohhot, Inner Mongolia 010018 P. R. China

**Keywords:** Home-made fermented dairy foods, *Streptococcus thermophilus*, Multilocus sequence typing, Population genetics, Food microbiology

## Abstract

**Background:**

*Streptococcus thermophilus* is a major dairy starter used for manufacturing of dairy products. In the present study, we developed a multilocus sequence typing (MLST) scheme for this important food bacterium. Sequences of 10 housekeeping genes (*carB*, *clpX*, *dnaA*, *murC*, *murE*, *pepN*, *pepX*, *pyrG*, *recA*, and *rpoB*) were obtained for 239 *S. thermophilus* strains, which were isolated from home-made fermented dairy foods in 18 different regions of Mongolia and China.

**Methods:**

All 10 genes of* S. thermophilus* were sequenced, aligned, and defined sequence types (STs) using the BioNumerics Software. The nucleotide diversity was calculated by START v2.0. The population structure, phylogenetic relationships and the role of recombination were inferred using ClonalFrame v1.2, SplitsTree 4.0 and Structure v2.3.

**Results:**

The 239 *S. thermophilus* isolates and 18 reference strains could be assigned into 119 different STs, which could be further separated into 16 clonal complexes (CCs) and 38 singletons. Among the 10 loci, a total of 132 polymorphic sites were detected. The standardized index of association (*I*_*A*_^*S*^ = 0.0916), split-decomposition and *ρ/θ* (relative frequency of occurrence of recombination and mutation) and *r/m* value (relative impact of recombination and mutation in the diversification) confirms that recombination may have occurred, but it occurred at a low frequency in these 10 loci. Phylogenetic trees indicated that there were five lineages in the *S. thermophilus* isolates used in our study. MSTree and ClonalFrame tree analyses suggest that the evolution of *S. thermophilus* isolates have little relationship with geographic locality, but revealed no association with the types of fermented dairy product. Phylogenetic analysis of 36 whole genome strains (18 *S. thermophilus*, 2 *S. vestibularis* and 16 *S. salivarius* strains) indicated that our MLST scheme could clearly separate three closely related species within the salivarius group and is suitable for analyzing the population structure of the other two species in the salivarius group.

**Conclusions:**

Our newly developed MLST scheme improved the understanding on the genetic diversity and population structure of the *S. thermophilus*, as well as provided useful information for further studies on the genotyping and evolutionary research for *S. thermophilus* strains with global diversity.

**Electronic supplementary material:**

The online version of this article (doi:10.1186/s12866-015-0551-0) contains supplementary material, which is available to authorized users.

## Background

*Streptococcus thermophilus* is a lactic acid bacteria species that is essential for the manufacturing of many types of fermented dairy products, occurring naturally as well as in commercial starter cultures [1]. Because *S. thermophilus* can produce large amounts of lactic acid, exopolysaccharides, and flavor compounds in fermentation processes, it is considered the second most important industrial dairy starter after *Lactococcus lactis* [2, 3]. Therefore, analyses of the genetic diversity, population structure, and phylogenetic relationships of *S. thermophilus* are important for understanding the evolution of ecological or biological properties of bacterial strains and for optimizing their industrial exploitation. Especially, *S. thermophilus* is genetically similar to *S. vestibularis* and *S. salivarius* in *S. salivarius* group. Several restriction- or PCR-based typing methods have been applied to the typing of *S. thermophilus* [4–8]. More recently, a powerful method for population genetic studies is multilocus sequence typing (MLST), which is based on sequencing of conserved housekeeping genes. The method provides faster and less expensive nucleotide sequence determination, and has become the gold standard for studying the evolution and population genetics of pathogenic microbes [9].

MLST involves the sequencing of a small number (at least seven) of housekeeping genes for each strain. The sequences of each fragment are assigned allele numbers for each locus. All allele numbers are combined to define the allelic profile of the strain and each different allelic profile is assigned as a sequence type (ST), which is used to describe the strain [10]. Because the typing is based on nucleotide sequence, MLST is primarily used for the exchange of electronic data between laboratories via the internet and provides accurate information on strain evolution [11, 12]. MLST has been used successfully in molecular epidemiology studies [13–15], and for identifying and typing lactic acid bacteria of various species of *Lactobacillus* [16–18], *Pediococcus* [19], *Enterococcus* [20], *Oenococcus* [21], and *Lactococcus* [22, 23]. The MLST scheme also reported for *S. thermophilus* analyzed the nucleotide variability at eight genetic loci of 27 *S. thermophilus* and the phylogenic relationship of two oral streptococci *S. salivarius* and *S. vestibularis* in the salivarius group [24].

In this study, 239 *S. thermophilus* isolated from different ecological sources and geographical areas and 36 whole genome strains (18 *S. thermophilus*, 2 *S. vestibularis* and 16 *S. salivarius* strains) were used to: (i) developed an effective MLST scheme for characterization of *S. thermophilus* and distinguish three closely related species in *S. salivarius* group; (ii) apply MLST to assess phylogenetic relationship and evolutionary characteristics of these strains; (iii) compare the genetic diversity of *S. thermophilus* from different type of dairy foods and geographic origin.

## Methods

### Bacterial isolates and DNA extraction

A total of 239 isolates of *S. thermophilus* were selected from Lactic Acid Bacteria Collection Centre (LABCC) of Inner Mongolia Agriculture University in China. Those included strains isolated from home-made fermented dairy foods (cow milk, yak milk, goat milk, mare milk and *Qula-*a kind of traditional cheese) in 6 provinces of China and 11 provinces and 1 city of Mongolia from 2005 to 2009. All these isolates were identified based on 16S rRNA gene sequencing, and some representative isolate were tested for gas production from glucose, salt tolerance (3.0 and 6.5 %), growth at different temperature (5, 10, 45, and 50 °C) and pH (3.0, 3.5, 4.0, and 7.0) in MRS broth [25–29]. The information of 239 *S. thermophilus* strains is listed in Additional file 1. Thirty six completed genome strains (18 *S. thermophilus*, 2 *S. vestibularis* and 16 *S. salivarius* strains) were chosen as reference strains for evaluate the novel MLST scheme (Table 1). Sequences of those completed genome strains were obtained directly from the NCBI genome database.

**Table 1:** 36 whole genome strains belonging to the salivarius group used in this study

Organism	Strains	GenBank Accession no.	Source	Location	Year
*Streptococcus salivarius*	HSISS1	GCA_000448645	Ileostomy effluent	Netherlands	2010
	HSISS4	GCF_000448685	Ileostomy effluent	Netherlands	2010
	JIM 8777	FR873482	Oral cavity	France	2004
	NCTC8618	CP009913	/	/	/
	UC3162	JYOY00000000	/	/	/
	CCHSS3	FR873481	Human blood	France	2002
	KB0005	JYOX00000000	/	/	/
	57.I	CP002888	Dental plaque	/	/
	SK126	ACLO00000000	Human skin	/	/
	M18	AGBV00000000	Oral swab from healthy volunteer	New Zealand	/
	K12	ALIF00000000	Saliva from healthy child	/	/
	NU10	JJMT00000000	/	/	/
	YU10	JJMS00000000	/	/	/
	C150	GCA_000187445	Human airway	/	
	PS4	AJFW00000000	Milk from a healthy woman	spain	2010
	HSISS3	GCF_000448605	Ileostomy effluent	Netherlands	2010
*Streptococcus vestibularis*	F0396	GCA_000180075	Oral cavity	England	1988
	ATCC 49124	GCA_000188295	Oral cavity	England	1988
*Streptococcus thermophilus*	M17PTZA496	CM002372	Fontina cheese	Italy	1996
	MTCC 5461	ALIL01000000	Fermented milk product (curd)	India	1984
	MTCC 5460	ALIK00000000	Fermented milk product (curd)	India	1984
	1F8CT	CM003138	Curd of “Grana Padano” cheese	Italy	2012
	TH1477	CM003135	Cow milk	Italy	2012
	TH1436	CM002370	Artisanal goat cheese from raw milk	Italy	2011
	CNRZ1066	CP000024	Commercial yogurt	France	1986
	LMG 18311	CP000023	Commercial yogurt	United Kingdom	1974
	MTH17CL396	CM002371	Fontina cheese	Italy	1996
	JIM 8232	FR875178	Raw milk	France	2002
	ND03	CP002340	Naturally fermented yak milk	China	2005
	TH985	CM003139	Buffalo mozzarella whey	Italy	2003
	TH982	CM003136	Buffalo moarella curd	Italy	2003
	LMD-9	CP000419	Starter for yogurt and mozzarella cheese	USA	/
	TH1435	CM002369	Artisanal goat cheese from raw milk	Italy	2011
	MN-ZLW-002	CP003499	Yogurt block	China	/
	DGCC 7710	AWVZ00000000	Commercial starter culture	/	/
	ASCC 1275	CP006819	/	/	/

All *S. thermophilus* strains were grown in 5.0 mL M17-broth (OXOID, CM0817B, Germany) supplemented with 5.0 g/L of lactose at 30 °C for 18–22 h, and then strains were harvested by centrifugation and cell pellets were used for DNA extraction. Total genomic DNA was extracted from cultures by using a previously reported method [30]. Purified DNA was diluted to a final concentration of 100 ng/μL for application.

### MLST loci selection

Ten housekeeping loci (*carB*, *clpX*, *dnaA*, *murC*, *murE*, *pepN*, *pepX*, *pyrG*, *recA* and *rpoB* gene) were selected for MLST analysis of *S. thermophilus* isolates based on the chromosome locations (preferably evenly separated across the entire genome), functions of the encoded proteins (preferably conserved and well characterized) and presence in all the strains as a single copy [21, 31]. The primers of ten genes were designed by Primer Premier 5.0 program (Premier Biosoft International) on the basis of known genome in *S. thermophilus* ND03 [32], and the information of primers is listed in Table 2.

**Table 2:** Genes and primers used for Multilocus sequence typing

Gene	Amplicon size (bp)	Application size (bp)	Gene function	PCR primer	Sequences (5’-3’)	Temperature
*carB*	652	499	*carB*amoyl phosphate synthetase	*carB*_primerF	AAGGCTACAGTGTTGTTCT	51 °C
				*carB*_primerR	GATAAGGTTTGCGTTGG	
*clpX*	768	630	ATP-dependent Clp protease subunit X	*clpX*_primerF	GCAGGTTATGTGGGTGA	52 °C
				*clpX*_primerR	TACTGGAGCAGCTTTCC	
*dnaA*	669	522	chromosomal replication initiator protein *dnaA*	*dnaA*_primerF	CACCAGGAGCAACTTAT	49 °C
				*dnaA*_primerR	CTTCAATCGGAATGAGA	
*murC*	592	451	UDP-N-acetyl muramate-alanine ligase	*murC*_primerF	TTTGTTTACGGTGAGGA	51 °C
				*murC*_primerR	CCACTTTGGCAGGTTTA	
*murE*	721	592	UDP-N-acetylmuramyl tripeptide synthase	*murE*_primerF	AACACCCTCAAGACAAA	48 °C
				*murE*_primerR	ACGAATACCTTAGCACC	
*pepN*	737	615	lysyl-aminopeptidase, aminopeptidase N	*pepN*_primerF	CGCTGAAGAGGGCGATAC	58 °C
				*pepN*_primerR	CTAACCAACGGCGGAGC	
*pepX*	664	524	X-prolyl dipeptidyl aminopeptidase	*pepX*_primerF	ACATCCCTGTTAGTCCTG	52 °C
				*pepX*_primerR	TCTCCCTCCATCTTGTG	
*pyrG*	736	624	CTP synthase	*pyrG*_primerF	CACTGAAGTTGGTGGGA	52 °C
				*pyrG*_primerR	CATACCGAGGCAGACAC	
*recA*	768	617	recombinase A	*recA*_primerF	AAAGAAGGTGGCATCGC	58 °C
				*recA*_primerR	ATCGTCCTCATCTAGCTCAAC	
*rpoB*	786	661	RNA polymerase beta subunit	*rpoB*_primerF	CATTACACGCACTACGG	47 °C
				*rpoB*_primerR	GATAACAGCATCCTCGA	

### PCR amplification and DNA sequencing

For each strain, the genomic DNA was used as a template for PCR amplification of MLST loci on the automatic thermal cycler (PTC-200, MJ Research, Waltham, MA). Thermal cycling conditions for PCR were: 94 °C for 5 min; 30 cycles of 94 °C for 1 min, corresponding temperature of each locus for 1 min, and 72 °C for 2 min; a final elongation step of 72 °C for 10 min. For each target, PCR mixture (50 μL) containing 150 ng of genomic DNA, 10 mM of each dNTP, 10 pmol of each primer, 2.5 U *Taq* polymerase in 1x PCR buffer (with 2.5 mmol/L Mg^2+^). PCR products were electrophoresed in a 1.2 % agarose gel. Sequencing of the PCR products was performed in Shanghai Majorbio Bio-pharm Technology Corporation. The same primers were used for PCR and sequencing on both DNA strands.

### MLST data analysis

For MLST analysis, forward and reverse sequences were trimmed, aligned, and analyzed using MEGA 6.0 software package (version 6.0, www.megasoftware.net) [33]. Definition of alleles using a nonredundant dataset and minimum spanning tree analysis were preformed using the BioNumerics Software (version 6.6, Applied-Maths, Sint Maartens-Latem, Belgium). Different allelic sequences (with at least one nucleotide difference) were assigned arbitrary numbers. For each of the ten MLST loci, a unique nucleotide sequence defined an allele. Unique allelic profiles, consisting of the allele numbers at each of the ten MLST loci, defined STs. The same ST was used for several strains when they shared the same allelic profiles. Groups of isolates with closely related allelic profiles have been called clonal complexes (CCs). STs were grouped into CC with the eBURST program [34] and located in the BioNumerics program.

START version 2.0 program [35] was used for calculating the number of polymorphic sites, mol% G + C content, *d*_*N*_/*d*_*S*_, and *I*_A_ and *I*_*A*_^*S*^ value for linkage analysis of the population as well as recombination testing. The nucleotide diversity (π) per site was calculated using Dnasp version 5.0 [36]. The split decomposition method was used to assess the degree of tree-like structure for alleles of each locus and all STs using SplitsTree 4.0 [37]. The phi test for recombination based on individual loci of the whole strain collection was also done with SplitsTree. The phylogenetic trees from the concatenated sequences (5718 bp) were constructed by the neighbor-joining method with a Kimura two-parameter distance model using MEGA 6.0. Bootstrap analysis with 1,000 replicates was performed.

The software Structure v2. 3 with linkage model [38] was used to identify the ancestral subpopulations and assign ancestry proportions for each isolate. The *K* value that generated the highest posterior probability was used as the probable number of ancestral populations. Three independent runs were performed for each value of the number of populations *K* ranging from 3 to 15. Each run consisted of 100,000 Markov Chain Monte Carlo (MCMC) iterations, of which the first 20,000 iterations were discarded as burn-in. The K value that generated the highest median posterior probability was used as the probable number of ancestral populations.

The evolutionary relationships amongst of *S. thermophilus* strains were inferred using ClonalFrame v1.2 [39]. Three independent runs of ClonalFrame were performed each consisting of 200,000 MCMC iterations, and a posterior sampling of 300,000 iterations. The prior iterations were discarded and model parameters were sampled in the second period of the run every 100th iteration thereafter, resulting in 3,000 samples from the posterior. The genealogy of the population was summarized and the robustness of the tree topology was evaluated by concatenating the posterior samples of the 3 runs to built-up a 50 % majority rule consensus tree using the ClonalFrame GUI. The ρ/θ and *r*/*m* was also accounted by ClonalFrame.

### Nucleotide sequence accession numbers

All MLST data of *S. thermophilus* in this study are available at http://pubmlst.org/sthermophilus/ [40]. Allele sequences of the ten MLST loci have been deposited in the GenBank database under accession numbers KF794203 to KF796592.

## Results

### Allelic profiles and sequence types

The 10 genes were successfully amplified for all strains and sequenced by bidirectional sequencing technology, and forward and reverse sequences were trimmed, aligned, and analyzed. Nucleotide positions of the sequences showing ambiguities were excluded from the analysis. Ultimately, the sequences of 10 housekeeping genes, ranging in size from 451 to 630 bp (Table 2), were used for MLST analysis.

Additional file 2 summarizes the allelic profiles of the 239 isolates and 18 *S. thermophilus* reference strains. A total of 119 different STs (ST1–ST119) were obtained; 83 STs corresponded to single isolates, 24 STs included 2–4 isolates, 8 STs included 5–9 isolates, and 4 STs included 11–15 isolates. The most represented STs were ST5 composed of 15 isolates (5.8 % of all isolates), ST2 (14 isolates, 5.4 %), ST79 (13 isolates, 5.1 %), and ST39 (11 isolates, 4.3 %). The breakdown by region was as follows: 140 strains from 12 different regions of Mongolia were identified as 67 STs, and 99 strains from 6 different provinces of China were assigned to 43 STs. Among the 119 STs identified in our collection, 14 STs were attributed to strains isolated in more than two provinces. The widest type was ST5, which was identified in strains isolated in as many as five different provinces across China and Mongolia. A similar distribution was observed for ST65, ST66, and ST91, which were identified in strains isolated from two provinces of China and Mongolia, respectively; the remaining ST strains were isolated in a specific province. Moreover, almost all genome strains were identified as a single ST, the exceptions being ND03, MN-ZLW-002, ASCC 1275, DGCC 7710, and LMD-9. Strain ND03 belonged to ST2, MN-ZLW-002 belonged to ST65, ASCC 1275 and DGCC 7710 belonged to ST66, and strain LMD-9 belonged to ST91.

### Nucleotide sequence variation at each MLST locus

We calculated the nucleotide diversity at each locus of all strains (Table 3). All loci were polymorphic and the number of polymorphic nucleotide sites varied between 8 (*recA* and *murC*) and 20 (*clpX*) suggesting a different rate of evolution. Between 7 (*recA*) and 18 (*clpX*) alleles were found for each locus. A total of 5718 nucleotides comprised 132 polymorphic sites. The mol% G + C content observed for different gene fragments varied from 36.89 % (*dnaA*) to 44.14 % (*pepN*) and was 39.1 % for the full *S. thermophilus* ND03 genome [32]. The nucleotide diversity per site (π) among the 10 genes varied from 0.0040 in *rpoB* to 0.0056 in *pepN*. The *d*_*N*_*/d*_*S*_ ratio of 10 loci ranged from 0.0311 (*pyrG*) to 0.2239 (*pepX*) and all were far less than 1 (Table 3), suggesting strong purifying selective pressure (negative selection) in these genes.

**Table 3:** Descriptive analysis of MLST data genetic variability at *Streptococcus thermophilus* loci

Gene	No. of		π^a^/site	G + C content (mol %)	*d*_*N*_/*d*_*S*_^b^
	alleles	polymorphic sites			
*carB*	10	10	0.0050	43.49	0.1439
*clpX*	18	20	0.0055	41.62	0.0789
*dnaA*	9	12	0.0054	36.89	0.0819
*murC*	9	8	0.0047	40.45	0.0360
*murE*	14	19	0.0048	41.64	0.0910
*pepN*	17	14	0.0056	44.14	0.1734
*pepX*	15	16	0.0053	41.97	0.2239
*pyrG*	12	12	0.0055	42.07	0.0311
*recA*	7	8	0.0043	39.08	0.0807
*rpoB*	13	13	0.0040	43.41	0.1046

a: Mean pairwise nucleotide difference per site

b: *d*_N_/*d*_S_ represents the ratio of nonsynonymous to synonymous substitutions

### Evidence for recombination in *S. thermophilus*

The multilocus linkage disequilibrium between MLST loci was measured using the index of association (*I*_A_) [41] and standardized index of association (*I*_*A*_^*S*^) [42]. To minimize linkage disequilibrium introduced by sampling bias or recent expansion of adaptive genotypes, only one strain from each ST was analyzed [22]. An *I*_*A*_ of 0.8242 (*P* = 0.000) and an *I*_*A*_^*S*^ of 0.0916 (*P* =0.000) for the 10 loci were obtained, which were greater than the value of 0 expected for a population at linkage equilibrium. This also shows the formation of an underlying clonal population structure of all the studied isolates. However, the low *I*_*A*_^*S*^ value confirms that recombination played a role in the evolution of the analyzed genes, and the split graphs of all ten loci and the concatenated sequences corroborated this. From the split graphs (Additional files 3 and 4), we can see that some parallelogram structures were formed for the *clpX*, *pepN*, *pepX*, *pyrG*, and *carB* genes indicating that these genes have undergone a little intergenic recombination during evolution (Additional file 3). In addition, the concatenated sequences of the 10 loci displayed a complex network-like structure (Additional file 4), suggesting that there have been several recombination events for these 10 loci based on the phi test (*P* = 2.108E-9). It is deduced that the selection of 5 genes with a more recombination history maybe contribute to the recombination events.

The linkage disequilibrium analysis and split graphs proved that recombination are existent in the evolution of 257 *S. thermophilus* strains. Then ClonalFrame was used to further assess the recombination or mutation play a leading role in the evolution of *S. thermophilus*. The *r*/*m* value (relative impact of recombination and mutation in diversification) and *ρ/θ* value (relative frequency of occurrence of recombination and mutation) were 0.0036 (95 % Credibility Interval 0.001 - 0.034) and 0.0034 (95 % Credibility Interval 0.001 - 0.031), respectively. These low values indicate that recombination occurred less frequently than mutation at these loci in the evolution of 257 *S. thermophilus*.

### Clonal complexes and minimum-spanning tree analysis

**Fig. 1:**
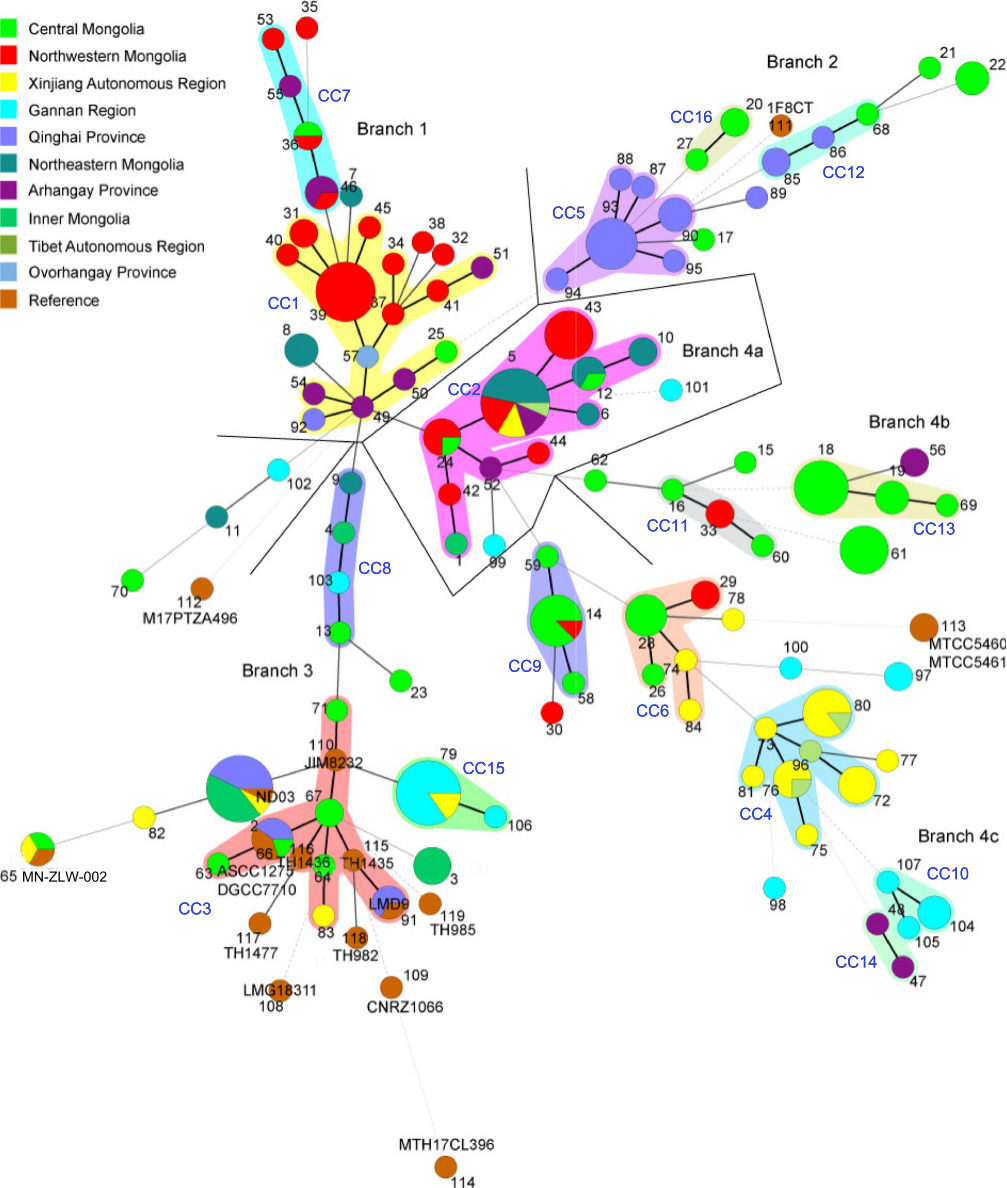
Minimum-spanning tree analysis of 239 *Streptococcus thermophilus* isolates and 18 reference strains based on the allelic profiles of 10 genes. Each circle corresponds to a sequence type (ST), and the circle size denotes the number of strains sharing the same ST. Colored zones between some groups of circles indicate that these profiles belong to the same clonal complex (CC). The strength of the link (bold, plain, or discontinuous) is related to the genetic similarity (number of common alleles) between profiles. Black line was drawn as boundaries of each branch

Assignment of STs to CCs by eBURST analysis divided 119 STs into 16 CCs (CC1–CC16) and 38 singletons (Additional file 2). The CC strains represented 73.2 % of all strains. The colored zones between some circle groups in Fig. 1 indicate profiles belonging to the same CC. The major CCs, CC1 (yellow zone), included 14 STs (corresponding to 25 strains) with ST49 identified as the ancestor genotype. CC1 comprised 24 strains isolated from seven different provinces in Mongolia, and only one strain isolated from Qinghai province in China (ST92). CC2 contained 36 isolates representing 10 STs (pink zone), with most of the strains isolated from Mongolia and 4 strains isolated from China. ST5 was identified as the ancestor ST of CC2. CC3 included 10 STs with ST67 identified as the ancestor genotype. 6 Mongolian isolates, 5 Chinese isolates, and reference strains JIM 8232, LMD-9, TH1435, TH1436, ASCC 1275, and DGCC 7710 comprise CC3 (red zone). CC4 consisted of 19 strains (light blue zone) and CC5 consisted of 15 strains (purple zone), all isolated from Xinjiang and Tibet, and Qinghai in China, respectively. All other CCs comprised less than 6 STs with a limited number of strains, and the strains in one CC were almost entirely from the same geographical location.

To analyze the relationship between the clusters, geographic origin, and the source dairy product among the 257 *S. thermophilus* isolates, allelic profile-based phylogenetic analysis was performed using a Minimum-Spanning Tree (MSTree) analysis with the BioNumerics v5.10 software. To facilitate analysis, Gansu and Sichuan province of China were grouped into Gannan region, and 11 provinces and one city in Mongolia were grouped into three larger regions (central, northwestern, and northeastern Mongolia) based on geographic position and environment. As shown in Fig. 1, strains of the same allelic profile were in the same circle, the size of which was proportional to the number of strains of that particular profile, and the different colors of the circles indicate different strain regions. The MSTree revealed four major branches and three minor branches. Branch 1 contained 39 strains from CC1, CC7 and 5 single STs. All strains were isolated from Mongolia except ST 92. Branch 2 contained three CCs (CC5, CC12, and CC16) and strain F8CT. The isolates in these branches were from Qinghai in China (19 strains) and central Mongolia (9 strains). Branch 3 contained three CCs (CC3, CC8, and CC15), with isolates from Xinjiang (6 strains), Gannan (13 strains), Qinghai (11 strains), Inner Mongolia (11 strains), central Mongolia (9 strains), and 13 reference strains. Regarding CC2 as the center, Branch 4 was divided into three minor branches (4a-c). Branch 4a included 38 strains of CC2, mainly from Mongolia (32 strains). Branch 4b, containing CC11 and CC13, contained 28 strains isolated from Mongolia. Branch 4c contained 55 strains of CC4, CC6, CC9, CC10, and CC14, most isolated from Xinjiang (20 strains), Tibet (3 strains), Gannan (9 strains), and central Mongolia (15 strains). Moreover, ST11, ST70, ST102, and ST122 do not belong to these four branches and are very different from ST 49. It is suggested that the phylogenetic relationships are more distant between these isolates compared to the others.

### Population structure and phylogenetic relationships

**Fig. 2:**
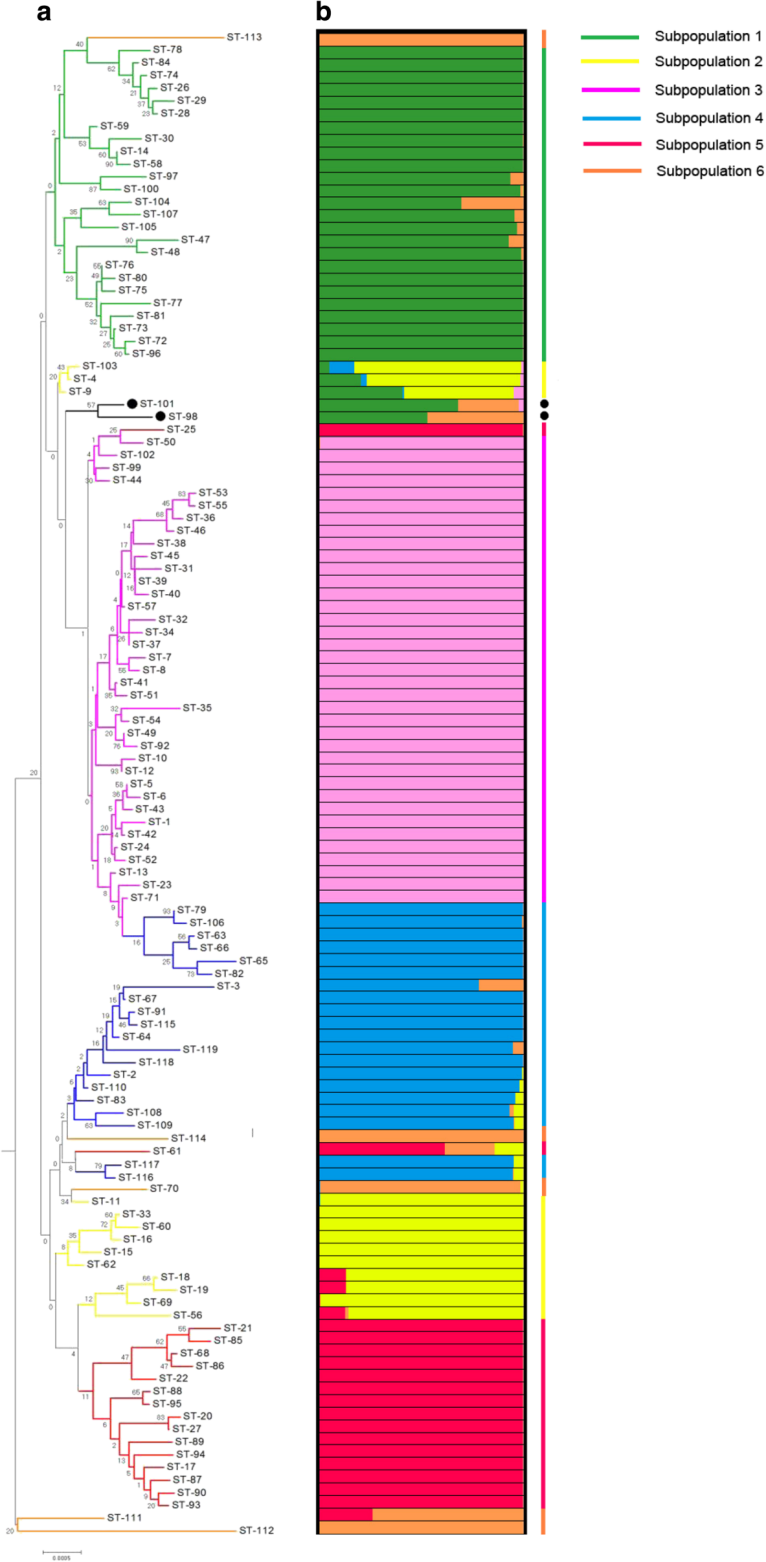
NJ tree and Ancestry of 119 *Streptococcus thermophilus* STs. **a** Neighbour-joining tree constructed from concatenated sequences of the 10 MLST loci. Bootstrap values are indicated for all branches. STs are colored according to their affiliation to one of the six ancestral subpopulations; admixed STs are in black. **b** Sources of ancestry of each unique ST from six ancestral subpopulations by Structure (assuming *K* = 6 populations and applying the linkage model). Six ancestral subpopulations are colored in Green (subpopulations 1), yellow (subpopulations 2), pink (subpopulations 3), blue (subpopulations 4), red (subpopulations 5) and orange (subpopulations 6). Each ST is represented by a single line with the ST designation at the top consisting of colored stacked bars that indicate the proportion of ancestry from each of six subpopulations

We attempted to statistically estimate the number of ancestral subpopulations (*K*) within the genetic population of the 119 *S. thermophilus* STs (257 isolates) using STRUCTURE with the linkage model. A maximal posterior probability of *K* = 6 was found in our sample, meaning that the genetic diversity of *S. thermophilus* can be divided into six ancestral subpopulations. For each ST, the average proportion of genetic material derived from each ancestral population is shown in Fig. 2b. The 119 STs fell into six distinct populations based on the major ancestral source of genetic diversity. Subpopulation 1 (green) contained 25 STs, 10 from Mongolia and 15 from Tibet, Xinjinag province, and Gannan in China. Subpopulation 2 (yellow) comprised 13 STs from Mongolia (23/25 strains). Subpopulation 3 (pink) consisted of 37 STs, mainly from Mongolia (72/79 strains), and subpopulation 4 (blue) consisted of 20 STs derived from central Mongolia strains and 13 reference strains. Subpopulation 5 (red) comprised 17 STs represented by isolates from central Mongolia (17/36) and Qinhai province in China (19/36). Subpopulation 6 (orange) contained 5 STs from five reference strains and one Mongolian strain. There was little admixture of ancestral sources among these six subpopulations, suggesting high homogeneity for the STs of each subpopulation. Moreover, ST98 and ST101 contained a high degree of admixture, which seemed to have acted frequently both as donors and as recipients of recombination exchanges.

The phylogeny of the 119 STs was analyzed by constructing a NJ tree from the concatenated sequences of 10 loci. As shown in Fig. 2a, the NJ tree revealed four major branches. The colored branches of the NJ tree are in accordance with the color of the groups identified in STRUCTURE. As shown in Fig. 2, we found that the STs of subpopulations 2 (yellow) and 4 (blue), 6 (orange) were distributed in differenced branches of the NJ tree. To examine any possible influences of recombination on tree topology, we inferred a clonal genealogy from our data using ClonalFrame, which takes recombination into account during tree building. The ClonalFrame tree (Additional file 5) showed all isolates fell into five lineages, A-E. Five subpopulations (1–5) identified in STRUCTURE (Fig. 2a) corresponded to the subpopulations shown in Additional file 5 and are represented by corresponding colors. However, subpopulation 6, found in STRUCTURE, did not constitute a lineage in the NJ analysis and ST114 (MTH17CL396) fell into lineage C. The other STs formed small clades at this level of analysis based on the ClonalFrame results. Based on the combined evidence from the STRUCTURE and ClonalFrame analyses, the 257 *S. thermophilus* strains formed five lineages during evolution.

### Phylogenetic analysis of *S. thermophilus* within the salivarius group

**Fig. 3:**
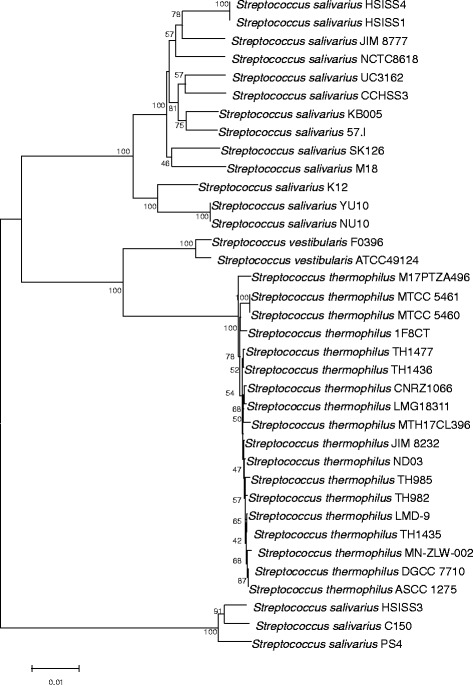
Phylogenetic tree of 18 *Streptococcus thermophilus*, 2 *S. vestibularis* and 16 *S. salivarius* strains, based on the concatenated sequences of 10 housekeeping genes

*S. thermophilus* belongs to the salivarius group, together with the two oral streptococci *S. salivarius* and *S. vestibularis*, and these three species are genetically very similar. A MLST scheme was created to analyze the phylogenetic relationships among these three species using five housekeeping genes (*ddlA*, *thrS*, *pyrE*, *sodA*, and *dnaE*) [43, 44]. *S. thermophilus* is the only nonpathogenic streptococcus originating from dairy products. Comparative genomic analysis found that the origin of genes acquired by *S. thermophilus* is suggested to be other LAB living in recurrent association with *S. thermophilus* in cheese or yogurt manufacture [45]. Therefore, we developed a new MLST scheme for *S. thermophilus* based on fragments of 10 housekeeping genes, which were used in a previous analysis of LAB. A phylogenetic tree was constructed using 36 whole genome strains, including 18 *S. thermophilus*, 16 *S. salivarius*, and 2 *S. vestibularis*, and the concatenated sequences of 10 loci (Fig. 3). It revealed four clear branches and the species *S. thermophilus*, *S. salivarius*, and *S. vestibularis* were supported by significant bootstrap values. The NJ tree also shows the existence of a fourth branch consisting of the three strains. In addition, 16 *S. salivarius* and 2 *S. vestibularis* were separated from each other, indicating that this MLST scheme is suitable for analyzing the population structure of all species in the salivarius group*.*

## Discussion

*S. thermophilus* is a key species involved in the acidification of milk and the development of texture in various fermented dairy products. However, few studies have characterized the diversity of the genetic population and the evolutionary scheme of this food bacterium from different ecological origins. To contribute to the characterization of the population structure of *S. thermophilus*, we utilized the MLST method to examine 239 strains isolated from different ecological sources and geographical areas.

MLST was first used for pathogenic microbes [10]. This technique is useful for elucidating the phylogenetic relationships and evolution of isolates. Traditional MLST schemes generally include only five to seven gene loci, the lengths of which range from 400 to 500 bp. This small number of gene loci and short length may lead to a decrease in information and unreliable results. Based on previous MLST studies of LAB and the *S. thermophilus* ND03 genome, 12 gene targets (*carB*, *clpX*, *dnaA*, *murC*, *murE*, *pepN*, *pepX*, *pyrG*, *recA*, *groEL*, *uvrC*, and *rpoB* gene) were first selected for MLST analysis in this study. Two loci (*groEL* and *uvrC*) could not be amplified from all examined strains and were removed. From these 10 gene loci, 239 *S. thermophilus* isolates and 18 genome strains were identified as 119 STs. The number of alleles per locus ranged from 7 to 18. When any gene locus with the lowest number of alleles was removed from the analysis, the number of STs decreased. Finally, an optimization of the MLST scheme based on these 10 housekeeping genes was chosen for analyzing *S. thermophilus*. Compared to previous MLST schemes [24], an average of 12 distinguishable alleles per locus among the 119 STs were identified, which is more than the average of 6–7 alleles observed from 27 *S. thermophilus* isolates in a previous study. Phylogenetic analysis of whole genome strains within the salivarius group showed that our method could clearly separate three closely related species and also confirmed the existence of an additional cluster. Our MLST scheme has strong typing discrimination for *S. thermophilus* and could be applied to the other two species in the salivarius group.

Analysis of synonymous and non-synonymous changes in the allele sequences of a locus can be used to determine whether it is subject to selection pressure. In our analysis, all loci displayed a *d*_*N*_*/d*_*S*_ ratio lower than 1, indicating a strong purifying selection preventing amino acid changes, which is a typical phenomenon for housekeeping genes and is desired in MLST schemes. The π of the 10 genes varied from 0.0040 in *rpoB* to 0.0056 in *pepN*. Previous reports on other lactic acid bacteria have ranged from 0.0004 to 0.0072 for *L. plantarum* [46], 0.0051 to 0.0096 for *L. delbruecki* [18], and 0.0038 to 0.0119 for *Leuconostoc mesenteroides* [47]. These results suggest that the examined *S. thermophilus* housekeeping genes are relatively conserved. Loss of gene function, recombination, and horizontal gene transfer (HGT) were proposed to contribute to the plasticity of the *S. thermophilus* genome [48, 49]. The low *I*_*A*_^*S*^ value (0.0916) and network structure of the split-graph in this study confirms that recombination may have occurred, while the low *ρ/θ* and *r/m* value indicate that recombination has not played a major role in the evolution of these loci. We infer that the discrepancy between our data and the literature may be related to the selected loci and *S. thermophilus* isolates.

The population structure within the sequences was analyzed using the linkage model in Structure. It is suggested that the six ancestral subpopulations in our isolates and some STs contain a high degree of admixture (Fig. 2). To examine any possible influences of recombination on tree topology, we inferred a phylogenetic tree using ClonalFrame. The ClonalFrame tree (Additional file 5) revealed that subpopulation 6 identified in STRUCTURE did not represent a true lineage, because this subpopulation is, in fact, a random mixture of strains that did not fall into one of the five true lineages and does not represent a real evolutionary lineage. However, the NJ tree contains four major branches, but they are not well supported because of low bootstrap values. The low bootstrap values are representative of the inherently incongruent phylogenetic signals in the NJ topology. It is suggested that the admixture of STs possibly contributed to the overall poor support values for the NJ topology [50]. Compared with the analytical tools in ClonalFrame and STRUCTURE, our data demonstrate that NJ analysis is not necessarily a suitable method for analyzing phylogenetic relationships between bacteria using MLST data.

One interesting application of the MLST technique was the identification of an association between strains and their origin. Previous studies on 40 *Lactobacillus casei* isolates using MLST demonstrated specificity to particular ecological niches [31]. However, the MSTree (Fig. 1) and ClonalFrame tree (Additional file 5) analyses in this study suggest that the evolution of *S. thermophilus* isolates have little relationship with geographic locality. Often, most strains from the same location clustered together, while the rest were dispersed across other clusters. The isolates from the same region are likely to have been exposed to similar environment selective pressures. For instance, Tibet and Xinjiang are located in western China, and have similar climate characteristics of drought and extensive sunshine, thus most isolates from those regions belong to the same lineage. Reference strain ND03 was isolated from Qinghai province in China, so it belongs to ST2 along with the 13 Qinghai isolates. In addition, it is interesting that the central Mongolian strains are distributed in several lineages. Central Mongolia includes the city of UlaanBaatar, the capital of Mongolia. Because of the traffic network in UlaanBaatar, trade contacts were more frequent with the other provinces of Mongolia and China. We deduced that fermented food exchange was a factor that may have contributed to the distribution of the central Mongolian strains, but the reference strains, especially the 12 European strains, have no strong relationship to the lineages and origins of the examined strains that could be identified, based either on the isolation of dairy sources or the sampling location. Furthermore, no significant associations between lineages and the type of dairy products were found in our collection of *S. thermophilus* (data not shown), which was probably due to an unequal number of isolates from different fermented products. A total of 182 *S. thermophilus* were isolated from fermented cow milk, whereas only 57 strains were from other fermented dairy products (3 isolates from fermented mare milk, 11 isolates from fermented goat milk, 5 isolates from *Qula*, and 38 isolates from fermented yak milk). As more isolates are collected from different countries and types of fermented products, further evidence may become available for the grouping of *S. thermophilus* compared to worldwide isolates, which would provide a strong indication of the factors that have affected its evolutionary history.

## Conclusions

MLST was used to study the genetic polymorphisms and evolutionary relationships of 239 isolates of *S. thermophilus* from different origins. Analysis of a geographically diverse and representative collection of isolates using MLST can provide a better understanding of *S. thermophilus* genome evolution, and provide information for future studies on the structure and genetic evolution of *S. thermophilus* globally.

## Additional files

Additional file 1:
**Bacterial strains used in this study.** (XLS 19 kb) Additional file 2:
**Allelic profiles of analyzed** ***Streptococcus thermophilus*** **strains.** (XLS 30 kb) Additional file 3:
**Split-decomposition of alleles for individual MLST loci of** ***Streptococcus thermophilus*** **strains.** (JPEG 250 kb) Additional file 4:
**Combined split-decomposition of alleles for the 10 MLST loci of** ***Streptococcus thermophilus*** **strains.** (JPEG 191 kb) Additional file 5:
**Clonal genealogy inferred by ClonalFrame from the 239** ***Streptococcus thermophilus*** **isolates and 18 reference strains.** Six subpopulations identified in Fig. 2b by Structure corresponded to lineages of the ClonalFrame clonal genealogy and have therefore been colored with the same colors as in Fig. 2. (PDF 1528 kb)
