# Supplementary figures and images for: Multilocus sequence typing of Streptococcus thermophilus from naturally fermented dairy foods in China and Mongolia
Source: BMC Microbiol. 2015 Oct 26;15:236. doi: 10.1186/s12866-015-0551-0 (PMC4620635; doi:10.1186/s12866-015-0551-0)

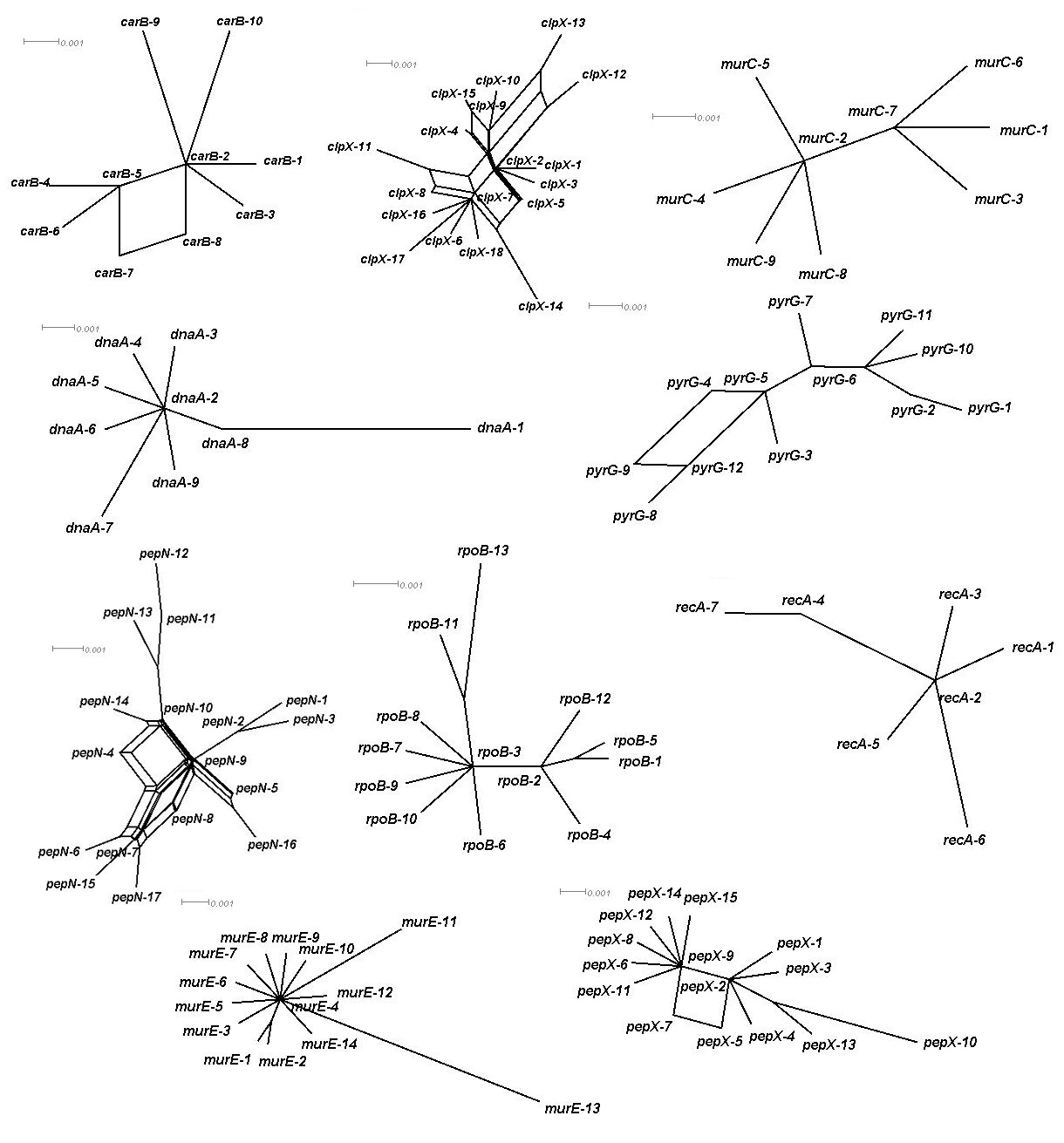

Supplement: Additional file 3: — Split-decomposition of alleles for individual MLST loci of Streptococcus thermophilus strains. (JPEG 250 kb) [file 12866_2015_551_MOESM3_ESM.jpeg]

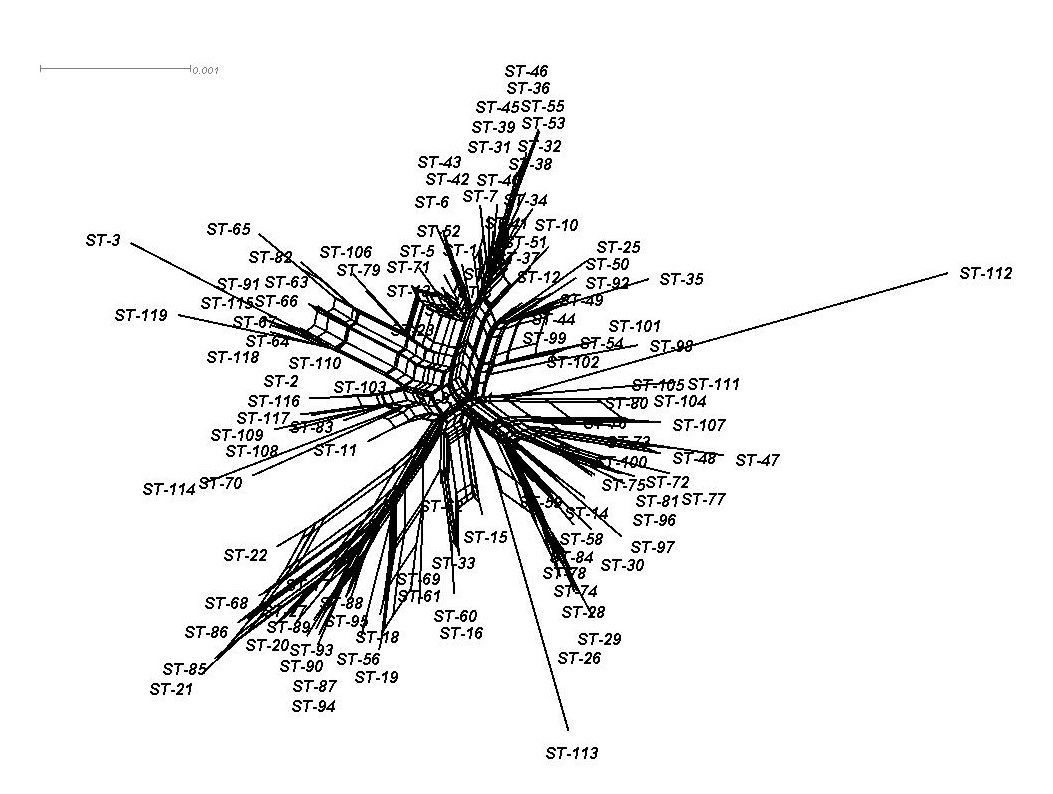

Supplement: Additional file 4: — Combined split-decomposition of alleles for the 10 MLST loci of Streptococcus thermophilus strains. (JPEG 191 kb) [file 12866_2015_551_MOESM4_ESM.jpeg]

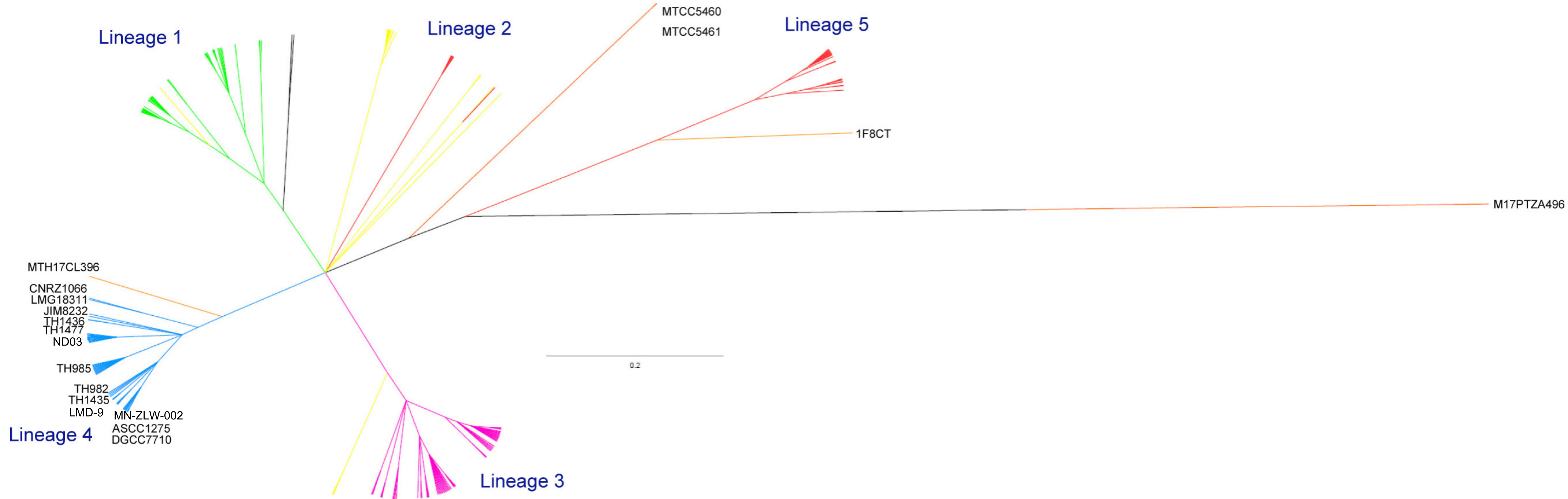

Supplement: Additional file 5: — Clonal genealogy inferred by ClonalFrame from the 239 Streptococcus thermophilus isolates and 18 reference strains. Six subpopulations identified in Fig. 2b by Structure corresponded to lineages of the ClonalFrame clonal genealogy and have therefore been colored with the same colors as in Fig. 2. (PDF 1528 kb) [file 12866_2015_551_MOESM5_ESM.pdf]
